# Prostaglandin E2 Secreted by Thyroid Cancer Cells Contributes to Immune Escape Through the Suppression of Natural Killer (NK) Cell Cytotoxicity and NK Cell Differentiation

**DOI:** 10.3389/fimmu.2018.01859

**Published:** 2018-08-09

**Authors:** Arum Park, Yunhee Lee, Mi Sun Kim, Young Ju Kang, Young-Jun Park, Haiyoung Jung, Tae-Don Kim, Hee Gu Lee, Inpyo Choi, Suk Ran Yoon

**Affiliations:** ^1^Immunotherapy Convergence Research Center, Korea Research Institute of Bioscience and Biotechnology, Daejeon, South Korea; ^2^Department of Functional Genomics, University of Science & Technology, Daejeon, South Korea; ^3^Department of Biochemistry, College of Pharmacy, Chungnam National University, Daejeon, South Korea; ^4^New Drug Development Center, OSONG Medical Innovation Foundation, Cheongju-si, South Korea

**Keywords:** prostaglandin E2, thyroid cancer, natural killer cell, immune escape, cytotoxicity, differentiation

## Abstract

Natural killer (NK) cells play important roles in immune surveillance. However, the tumor microenvironment suppresses NK cell function and allows cancer cells to evade immune detection. In this study, we investigated whether the thyroid cancer cell microenvironment has this effect on NK cells. We found that prostaglandin (PG) E2 produced by thyroid cancer cells suppressed the cytolytic activity of NK cells by inhibiting the expression of the natural cytotoxicity receptors NKp44 and NKp30 and the death receptor tumor necrosis factor-related apoptosis-inducing ligand. PGE2 and cyclooxygenase-2 were highly expressed in thyroid cancer cells; moreover, anaplastic thyroid cancer cells released higher amounts of PGE2 than the papillary subtype, which was associated with suppression of NK cell-inducing nuclear factor-κB and mitogen-activated protein kinase/extracellular signal-regulated kinase pathways *via* PGE2 receptor (EP) 2 and EP4 expressed on the NK cell surface. In addition, PGE2 inhibited the functional maturation of NK cells and reduced their cytotoxicity against target cells. These results indicate that PGE2 promotes thyroid cancer progression by inhibiting NK cell maturation and cytotoxicity. Thus, therapeutic strategies that target PGE2 in thyroid cancer could potentiate the immune response and improve patient prognosis.

## Introduction

Natural killer (NK) cells are lymphocytes that have natural cytotoxicity in antiviral and antitumor responses of the innate immune system (1, 2). NK cells target tumor cells through various mechanisms, including granule exocytosis, death receptor-mediated killing, and interferon (IFN)-γ secretion; interactions with target cells are mediated *via* activating and inhibitory receptors (3–6). Typical activating receptors include NK group 2, member D (NKG2D), and natural cytotoxicity receptors NKp44, NKp46, and NKp30. NK cells use perforin and granzyme B to penetrate into target cells and induce their death. Activated NK cells also secrete IFN-γ to stimulate other immune cell types and activate an immune response.

Various types of cancer cell express ligands that are recognized by NK cells and stimulate their cytotoxic activity. NKG2D recognizes UL16-binding protein and major histocompatibility complex class I polypeptide-related sequence A/B expressed on the surface of cancer cells (7, 8); proliferating cell nuclear antigen binds to NKp44 (9), while B7-H6 molecule and B cell lymphoma 2-associated athanogene 6 are recognized by NKp30 (10). These ligands are more highly expressed in tumor cells as compared to normal cells.

The immune system eliminates cancer cells under normal conditions; however, these cells create a defined microenvironment by releasing immunosuppressive cytokines, growth factors, and enzymes that protect them from immune surveillance mechanisms (11–15). For example, cancer cells produce small molecules, such as indoleamine 2,3-dioxygenase (IDO), transforming growth factor (TGF)-β, interleukin (IL)-10 and -6, and prostaglandin (PG)E2, which suppress immune cell activity (16). PGE2 is a small lipid molecule upregulated in various cancers that induces cyclooxygenase (COX)-2 activity (16); its expression levels are associated with cancer type or stage (17, 18).

Thyroid cancer is classified into papillary, follicular, medullary, and anaplastic types. Most thyroid cancers are of the papillary type and are easily treated due to their slow progression. In contrast, anaplastic thyroid cancer (ATC) is difficult to control due to rapid growth of the cancer cells (19–21), which may be associated with immune evasion and NK cell suppression. Therefore, clarifying the mechanism underlying NK cell suppression by thyroid cancer cells can provide a basis for the development of more effective therapeutic strategies.

To address this issue, in the present study we investigated the effect of PGE2—a factor present in thyroid cancer cell culture supernatant—on NK cell activity. Our results demonstrate that PGE2 reduced NK cytotoxicity by inhibiting the expression of specific receptors on the NK cell surface. In the presence of PGE2, NK cells remained in functionally immature state with low cytotoxicity. In addition, ATC exhibiting poor prognosis released higher amounts of PGE2 than the papillary type.

## Materials and Methods

### Ethics Statement

This study was approved by the Institutional Review Board of the Asan Medical Center according to the Bioethics and Safety Act and the Declaration of Helsinki. Each participant provided written, informed consent.

### NK Cell Isolation and Culture

Human NK cells were isolated from the peripheral blood of healthy donors using RosetteSep (Stem Cell Technologies, Vancouver, BC, Canada)—which depletes cluster of differentiation 3^+^ T cells and red blood cells—followed by CD56 magnetic beads (Miltenyi Biotec, Bergisch Gladbach, Germany). The cells were cultured in α Minimal Essential Medium (Welgene, Gyeongsan, Korea) with IL-15 (30 ng/ml), IL-21 (30 ng/ml), and 10^−6^ M of hydrocortisone (HC; Stem Cell Technologies, Canada). To investigate the effect of PGE2 on NK cell toxicity, the cells were cultured for 48 h with either control medium or thyroid cancer cell culture supernatant at 1/4 dilution.

### Differentiation of NK Cells From Hematopoietic Stem Cells (HSCs)

Hematopoietic stem cells were isolated from umbilical cord blood (CB) cells of pregnant women using the CD34 Micro Bead kit (Miltenyi Biotec). CD34 + HSCs were differentiated into precursor (p)NK cells in pNK medium containing IL-7 (5 ng/ml), stem cell factor (30 ng/ml), FMS-like tyrosine kinase 3 ligand (50 ng/ml), and 10^−6^ M HC in MyeloCult H5100 (Stem Cell Technologies) for 14 days. The pNK cells were induced to differentiate into mature (m)NK cells in mNK medium containing IL-15 (30 ng/ml), IL-21 (30 ng/ml), and HC in MyeloCult H5100 for 14 days. All cytokines used for NK cell differentiation were purchased from PeproTech (Rocky Hill, NJ, USA). Fresh PGE2 was added when the culture medium was changed during NK differentiation.

### Thyroid Cancer Cell Lines and Thyroid Cancer Cell Supernatant

Papillary thyroid cancer (PTC) cell lines, including TPC-1, BCPAP, and ATC cell lines including FRO, 850-5C were purchased from American Type Culture Collection (ATCC, Manassas, VA, USA). BCPAP, FRO, and 850-5C cells were cultured in RPMI including 10% FBS (Welgene). TPC-1 cells in DMEM including 10% FBS at 37°C incubator. Thyroid cancer cells were cultured in 1 × 10^6^ cells per 100 mm^2^ dish for 72 h with or without COX-2 inhibitor. COX-2 inhibitor, NS398 (Sigma-Aldrich, St. Louis, MO, USA) treated at 1 and 5 μM.

### mRNA Analysis by Quantitative RT-PCR

Total RNA was extracted from cells using RNeasy^®^ Plus Mini Kit from QIAGEN (Hilden, Germany) and synthesis of cDNA was performed using kit (TOYOBO, Osaka, Japan) and checked by real-time PCR using SYBR^®^ Premix (Takara Co., Shiga, Japan). The primer sequences were as follows; for *EP1* 5-TGCCCATCTTCTCCATGACG-3 and 5-CCACGAACAGCAGGAAGGT-3; for *EP2* 5-CCTTGGGTCTTTGCCATCCT-3 and 5-GACCTCAAAGGTCAGCCTGT-3; for *EP3* 5-CCCTTCCGTTGGTTCTTGGA-3 and 5-AGCTCGCAAGTGTTAGTGGA-3; for *EP4* 5-AATTTGCTTCCAGGTGTGCC-3 and 5-CCCTGTGAAGAGTCTGAGGT-3; for *vav1* 5-TGCTTCAAGTCTCTGGACACCAC-3 and 5-TCTCGGGCGCAGAAGTCTA-3; for *c-Jun*, 5-GAGCGGACCTTATGGCTACA-3 and 5-CCGTTGCTGGACTGGATTAT-3; for *c-Fos*, 5-AAGGAGAATCCGAAGGGAAA-3 and 5-GTGAGCTGCCAGGATGAACT-3; for *PU.1*, 5-CAGCTCTACCGCCACATGGA-3 and 5-TAGGAGACCTGGTGGCCAAGA-3; for *IKAROS*, 5-GATGAAGAGAATGGGCGTGC-3 and 5-TTCTCTCCCGAGGCATCAAG-3; for *T-bet*, 5-TGGGTGCAGTGTGGAAAGG-3 and 5-CAAATGAAACTTCCTGGCGC-3; and for *Ets-1*, 5-ACCACAGACTTTGAGGGAAGC-3 and 5-AGCTCATTCTGCTCTCAGCAC-3.

### Flow Cytometry Analysis

Natural killer cells were stained with appropriate antibodies for further analysis. For surface staining, FITC-anti-CD158a (Clone HP-3E4), PE-anti-CD158b (Clone CH-L), APC-anti-CD56 (Clone B159), FITC-anti-NKp46 (Clone 9-E2), PE-anti-NKp44 (Clone p44-8), FITC-anti-CD16 (Clone B73.1), PE-anti-NKp30 (Clone p30-15), FITC-anti-CD158e (Clone DX9), PE-anti-NKG2D (Clone 1D11), APC-anti-CD107a (Clone H4A3) PE-anti-FASL (Clone NOK-1), and PE-anti-TRAIL (Clone RIK-2) were used. APC-anti-IFN-γ (Clone B27) was stained by cell fixation/permeabilization kits (BD Bioscience, Franklin Lakes, NJ, USA). Antibodies for immune-staining were purchased from BD Bioscience. The data of samples were acquired by FACS Canto II (BD Bioscience) and analyzed using software Flow Jo (Tree Star, Inc., Ashland, OR, USA).

### Western Blot Analysis

Thyroid cancer cells (5 × 10^5^ per sample) were incubated at 37°C for 48 h in culture medium and then lysed using radioimmunoprecipitation assay cell lysis buffer (GenDEPOT, Katy, TX, USA) containing protease and phosphatase inhibitors. Samples were incubated with primary antibodies against β-actin (Santa Cruz Biotechnology, Santa Cruz, CA, USA) or COX-2 (Cell Signaling Technology, Danvers, MA, USA). NK cells were cultured with 100 and 500 ng/ml PGE2 at 37°C for 24 h and then lysed in lysis buffer. Primary antibodies against p65 (D14E12), phosphorylated (p-) p65 (93H1), extracellular signal-regulated kinase (ERK) (137F5), and p-ERK (D13.14.4E) were purchased from Cell Signaling Technology.

### Evaluation of NK Cell Cytotoxicity

Cytotoxicity was evaluated by calcein-AM release assay (22). Briefly, K562 target cells were labeled with calcein (Invitrogen, Carlsbad, CA, USA) for 1 h. Calcein-labeled target cells (1 × 10^4^ cells) and serially diluted effector cells were then co-cultured in 96-well round-bottom plate for 4 h. “Maximum release” was simulated by adding 2% Triton X-100 to the target cells, and “Spontaneous release” was simulated by adding culture media to the target cells. The calcein released into the supernatant was measured using a multi-mode microplate reader (Molecular Devices, San Jose, CA, USA). The percent specific lysis was calculated according to the formula [(test release − spontaneous release)/(maximum release − spontaneous release)] × 100.

### Cytokine Measurement by Enzyme-Linked Immunosorbent Assay (ELISA)

Cytokines, including interleukin(IL)-2, IL-4, IL-5, IL-6, IL-10, IL-10, IL-12, IL-13, IL-17A, tumor necrosis factor-α, transforming growth factor (TGF)-β, interferon(IFN)-γ, and granulocyte-colony stimulating factor were evaluated in culture supernatant using specific ELISA array kit from QIAGEN (Germany). Soluble PGE2 was measured by specific ELISA kit purchased from ENZO life sciences (Farmingdale, New York, NY, USA).

### Statistical Analysis

Statistical significance was evaluated by Student *t*-test. A *P* value of less than 0.05 (*), less than 0.01 (**), or less than 0.001 (***) was considered statistically significant.

## Results

### Culture Supernatant of Thyroid Cancer Cells Suppresses NK Cell Cytotoxicity

**Figure 1:**
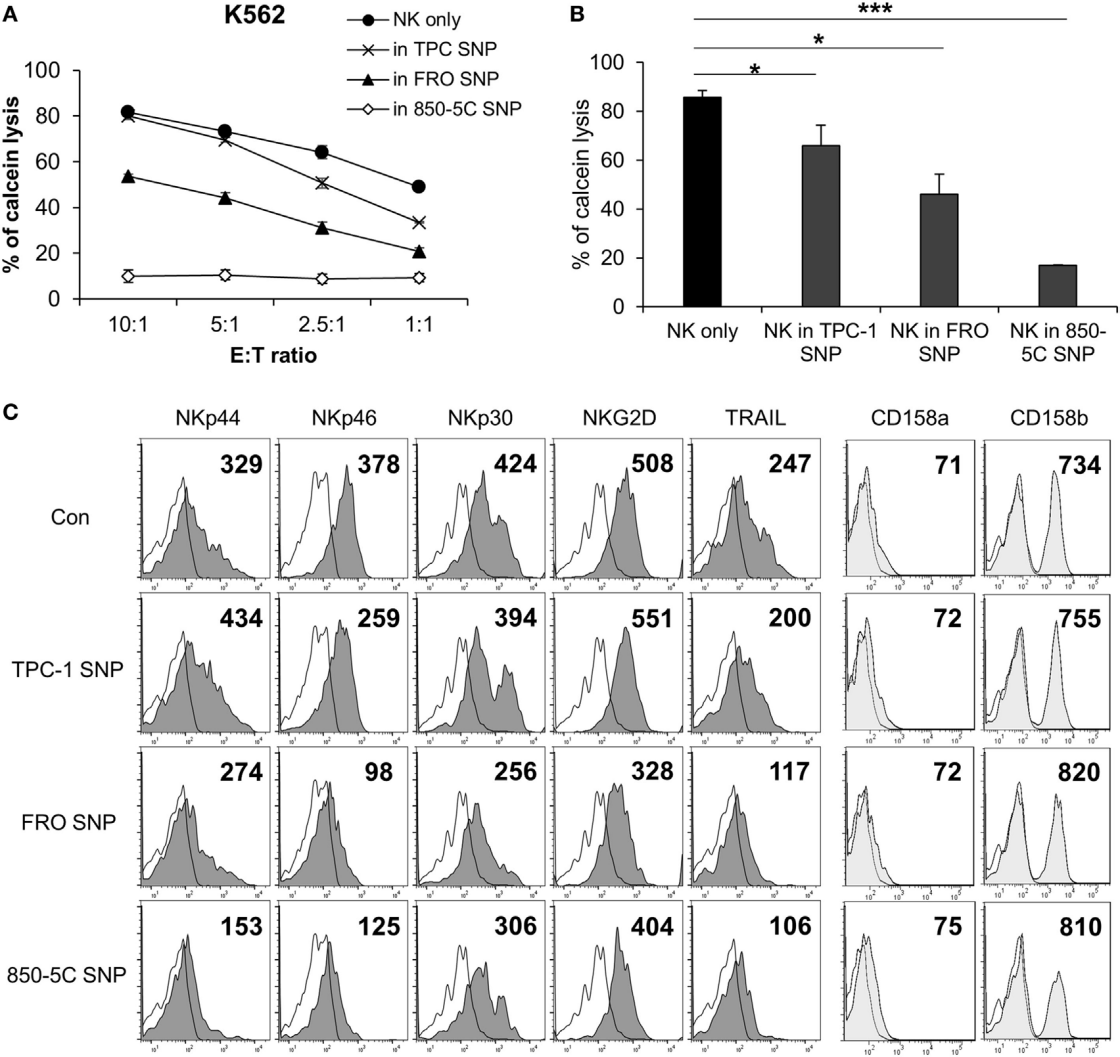
Culture supernatants of thyroid cancer cells inhibit natural killer (NK) cell cytotoxicity. **(A)** NK cells were cultured with IL-2 either in control medium (circle) or in medium containing culture supernatant of thyroid cancer cells including TPC-1 (cross), FRO (triangle), and 850-5C (diamond) for 48 h. The cytotoxicity of the NK cells was assessed relative to K562 cells at different E:T ratio as described in Section “Materials and Methods.” Bars represent mean ± SD from three experiments. **(B)** Percent cytotoxicity by NK cells treated with culture supernatants of thyroid cancer cells relative to NK cells grown in control medium. K562 cells were used as targets and the effector:target (E:T) ratio was bar which represent mean ± SD of three independent experiments. Statistical analysis was performed using the paired two-tailed Student’s *t*-test. *P < 0.05 and ***P < 0.001. **(C)** Expression of NK activating receptors and death receptors (profiles filled with dark gray), inhibitory receptors (profiles filled with light gray), and isotype controls (white profiles) was analyzed by flow cytometry. Numbers indicate mean fluorescence intensity. *Y*-axis shows cell counts.

To investigate whether the cytolytic activity of NK cells is affected by the thyroid cancer microenvironment, the cells were cultured in the presence or absence of supernatants from papillary (TPC-1) and anaplastic (FRO and 850-5C) thyroid cancer cell cultures along with human IL-2 for 48 h; the cytotoxicity of NK cells was measured against K562 cells. The cytolytic activity of NK cells was markedly reduced in the presence of culture supernatants of all thyroid cancer cells tested (Figures 1A,B) with the supernatant of ATC cell cultures showing a more potent effect that than of TPC-1 cell cultures. The thyroid cancer cell culture supernatant decreased the expression of the activating receptors NKp46, NKp44, NKp30, and NKG2D in NK cells (Figure 1C; Figure S1A in Supplementary Material) as well as the level of TRAIL, which binds to death receptors expressed by cancer cells. Anaplastic cancer cell (FRO and 850-5C) culture supernatants significantly reduced the expression of NK activating receptors and TRAIL compared to papillary cancer cell (TPC-1) supernatant. However, none of the thyroid cancer cell culture supernatants altered the expression levels of NK cell inhibitory receptors such as killer cell immunoglobulin-like receptor (KIR)2DL1 and KIR2DL3 (Figure 1C). These results indicate that soluble factors secreted by thyroid cancer cells inhibit the cytolytic activity of NK cells by suppressing the expression of NK activating receptors.

### PGE2 in Culture Supernatant of Thyroid Cancer Cells Inhibits NK Cell Cytotoxicity

**Figure 2:**
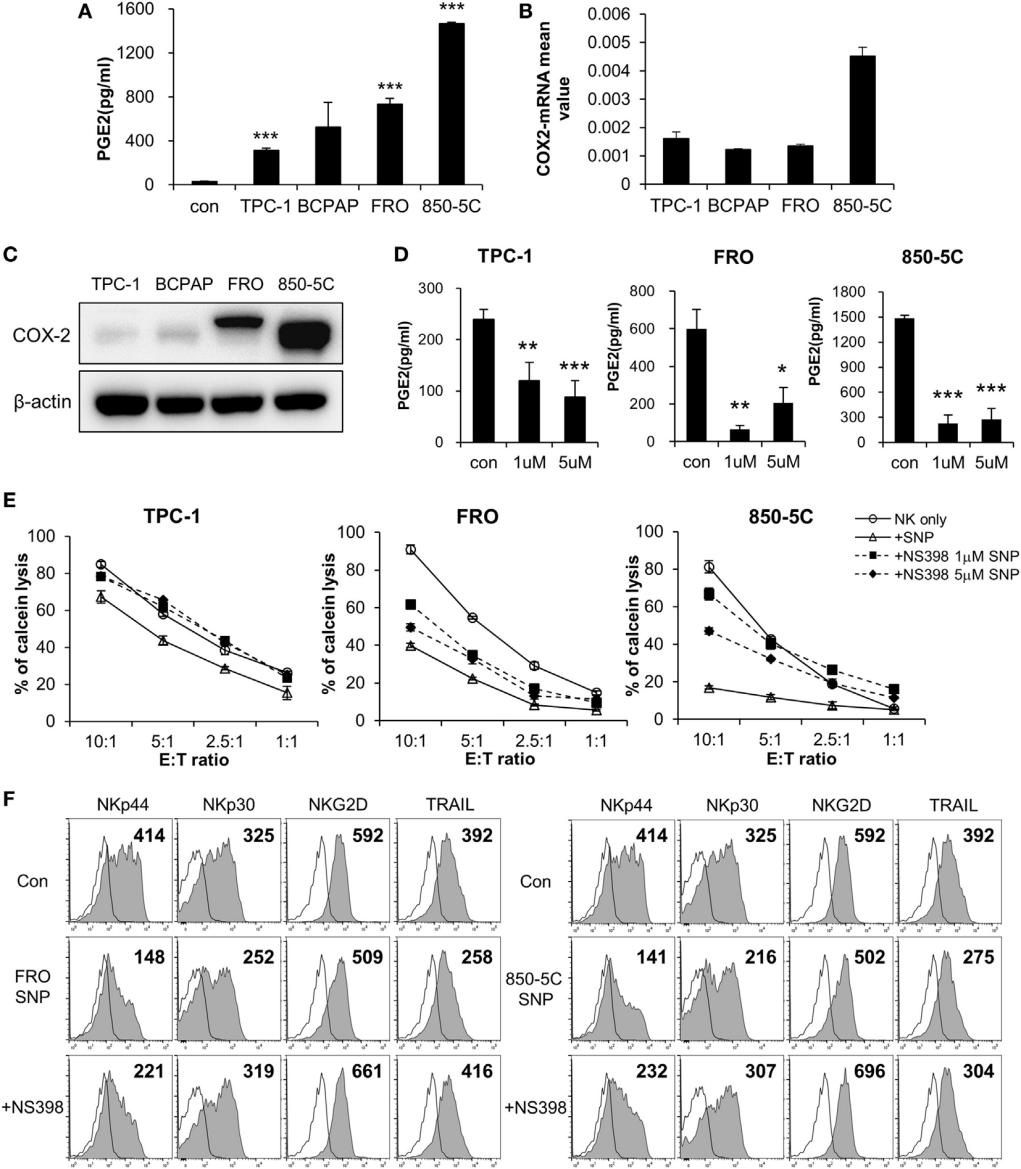
COX2 inhibition with NS-398 blocks PGE2 production and restores natural killer (NK) cell activity reduced by thyroid cancer. **(A)** Concentrations of PGE2 in culture supernatants from thyroid cancer cells, as measured by enzyme-linked immunosorbent assay (ELISA). Bars represent mean ± SD of three independent experiments. Statistical analysis was performed using the paired two-tailed Student’s *t*-test. **(B)** mRNA expression of COX-2 in thyroid cancer cells, as determined by real-time PCR. Glyceraldehyde 3-phosphate dehydrogenase served as an internal control. **(C)** Expression of COX-2 in thyroid cancer cells detected by Western blotting; β-actin served as a loading control. **(D)** PGE2 concentrations in supernatants of thyroid cancer cell cultures with or without NS398 for 72 h, as determined by ELISA. Bars represent mean ± SD of TPC-1 *n* = 6, FRO, and 850-5C *n* = 3. Statistical analysis was performed using the paired two-tailed Student’s *t*-test. *P < 0.05, **P < 0.01, and ***P < 0.001. **(E)** NK cells cultured with IL-2 in the control medium (circle) or in culture supernatant (triangle) or culture supernatant + NS398 for 48 h; cytotoxicity against K562 cells was evaluated as a control. NS398 was administered at 1 µM (square) or 5 µM (diamond). Bars represent mean ± SD of three independent experiments. **(F)** Expression of NK receptors NKp44, NKp30, and TRAIL (filled profiles) and isotype controls (white profiles) analyzed by flow cytometry. Numbers indicate mean fluorescence intensity. *Y*-axis shows cell counts.

To identify the factor(s) in the culture supernatant of thyroid cancer cells that inhibit(s) the cytolytic activity of NK cells, we measured the levels of various soluble factors using an ELISA array kit (Figure S2 in Supplementary Material) and PGE2 ELISA kit. PGE2 levels were elevated in the culture supernatants, and were higher in those of ATC cells (FRO and 850-5C) as compared to PTC cells (TPC and BCPAP) (Figure 2A; Figure S3 in Supplementary Material). This suggests that PGE2 production is related to the progression of thyroid cancer. To investigate this possibility, we evaluated the expression of the PGE2-generating enzyme COX-2 and found that it was higher in anaplastic as compared to PTC cells (Figure 2B). Strikingly, COX-2 protein was detected only in FRO and 850-5C cells, implying that PGE2 production is associated with the development of ATC (Figure 2C). To confirm the effect of PGE2 produced by thyroid cancer cells on NK cells, thyroid cancer cells were cultured in the presence or absence of the COX-2 inhibitor NS398 to block PGE2 production. NS398 treatment inhibited PGE2 production (Figure 2D). NK cells were then cultured in medium containing supernatant of thyroid cancer cell cultures for 48 h, and NK cytolytic activity was measured. In the absence of NS398, cytolytic activity and expression of activating receptors were decreased as compared to NK cells without treatment. However, NK cells grown in medium containing culture supernatant from thyroid cancer cells treated with NS398 showed increased cytolytic activity as compared to those grown in medium containing supernatant of cancer cell cultures without NS398 treatment (Figure 2E). Blocking PGE2 production in thyroid cancer cells with COX-2 inhibitor restored NK cell cytotoxicity, with a more pronounced effect observed in anaplastic cancer cells. In addition, the decreased levels of the activating receptors NKp44 and NKp30 in NK cells exposed to untreated cancer cell supernatants were also restored by the culture supernatant of cells treated with NS398 (Figure 2F; Figure S1B in Supplementary Material). These results indicate that PGE2 secreted by thyroid cancer cells suppresses NK cell cytotoxicity by inhibiting the expression of NK activating receptors.

### PGE2 Influences NK Cell Activity Through EP2 and EP4 Receptors

**Figure 3:**
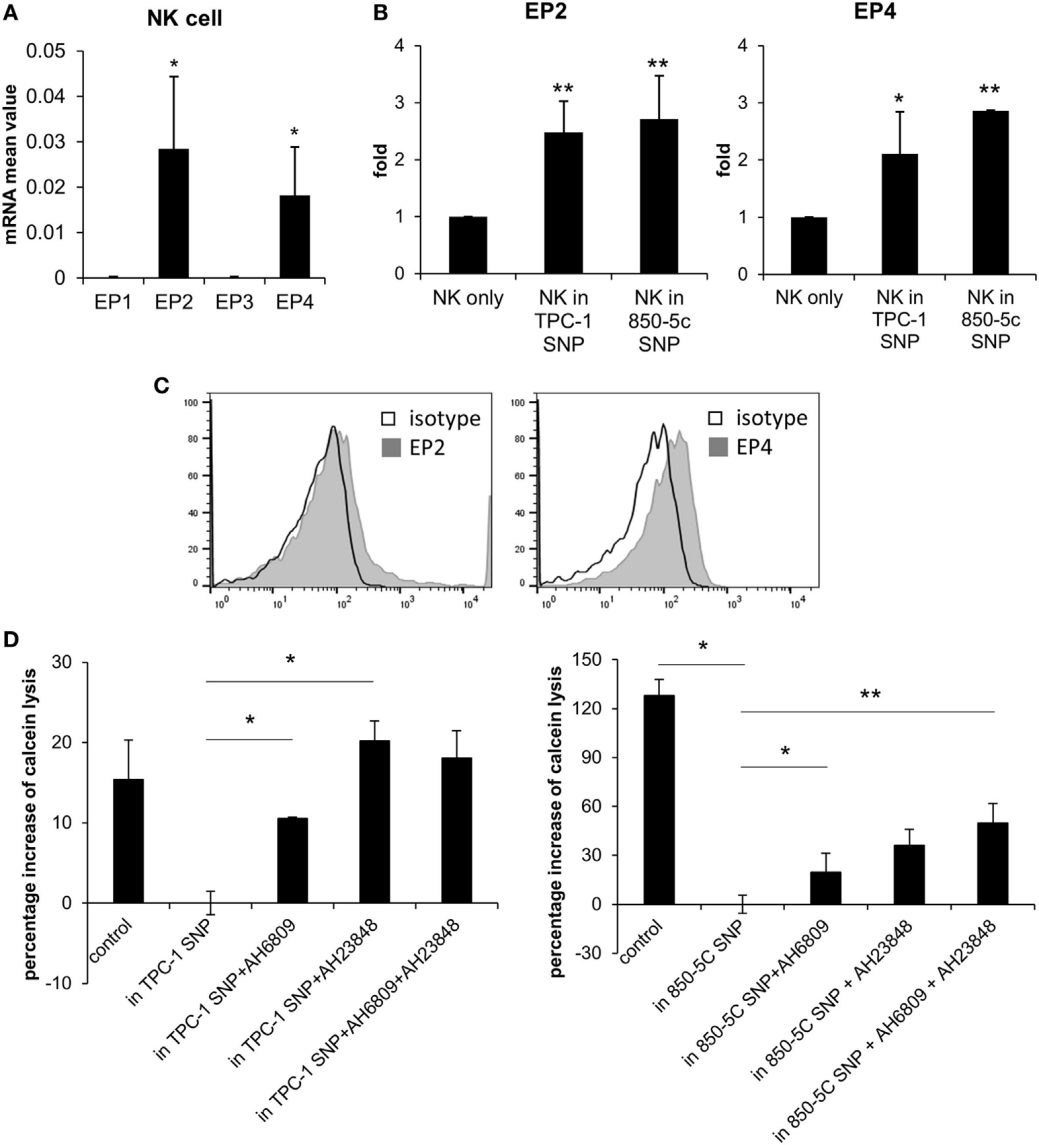
PGE2 inhibits natural killer (NK) cell activity *via* EP2 and EP4 receptors. **(A)** mRNA level of EP receptors on NK cells and **(B)** changes in EP2 and EP4 expression after culturing with thyroid cancer cell supernatants for 48 h, as determined by real-time PCR. Glyceraldehyde 3-phosphate dehydrogenase served as an internal control. **(C)** Histograms representing EP2 and EP4 expression in NK cells detected by flow cytometry. **(D)** NK cells were cultured with IL-2 for 48 h in control medium or in culture supernatant without or with AH6809 (EP2 receptor antagonist) or AH23848 (EP4 receptor antagonist). Cytotoxicity was assessed against K562 cells using calcein-AM. Effector to target ratio was 5:1. The graph was represented by calculating the percentage increase based on NK cells treated with cancer cell supernatant. Bars represent mean ± SD of three independent experiments. *P < 0.05 and **P < 0.01 vs. NK cells cultured in thyroid cancer cell supernatants Statistical analysis was performed using the paired two-tailed Student’s *t*-test.

We investigated the mechanism by which PGE2 inhibits NK cell activity, including the receptors involved. PGE2 is internalized by a variety of cell types *via* four PGE2 receptors (EP1–4). EP2 and EP4 are related to immune function (23, 24). We evaluated the expression of EP receptors on NK cells and detected EP2 and EP4 but not EP1 or EP3 transcripts in NK cells activated by IL-2 (10 ng/ml) (Figure 3A). EP2 and EP4 levels were upregulated in cells were cultured with thyroid cancer cell supernatants (Figure 3B). EP2 and EP4 proteins were also detected on the NK cell surface by immunolabeling and fluorescence-activated cell sorting (Figure 3C). To confirm that PGE2 acts on NK cells *via* EP2 and EP4, the cells were cultured in thyroid cancer cell supernatants in the presence or absence of the EP2 antagonist AH6809 (20 µM) and/or the EP4 antagonist AH23848 (20 µM) (each at 10 µM) to block EP receptor signaling. NK cells treated with the supernatant of thyroid cancer cells showed reduced cytotoxicity as compared to untreated control NK cells. However, this effect was reversed by blocking EP receptors even though there were differences in recovery extent. Combined treatment with AH6809 and AH23848 had no synergistic effect compared to single treatment with thyroid cancer cell supernatant (Figure 3D). These results imply that PGE2 in the supernatant of thyroid cancer cells inhibits the activity of NK cells through EP2 and EP4 receptors.

### PGE2 Inhibits NK Cell Activation *via* Regulation of ERK and Nuclear Factor (NF)-κB Pathways

**Figure 4:**
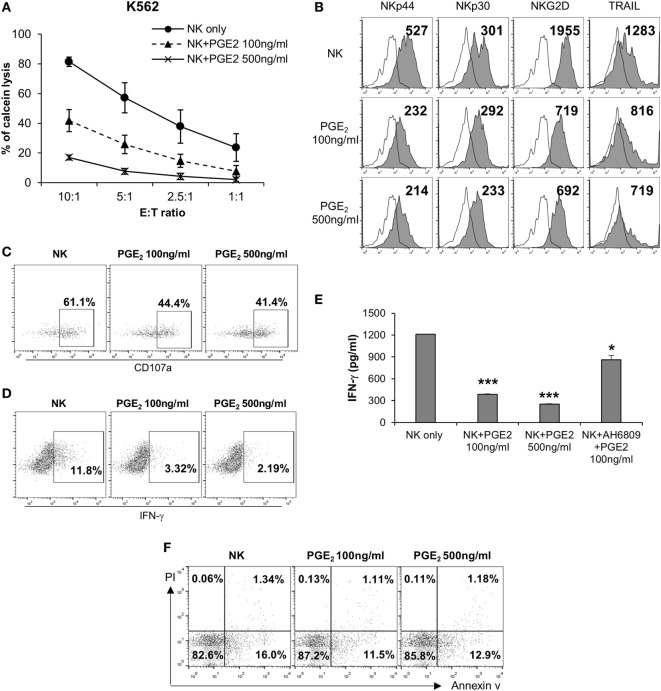
PGE2 inhibits natural killer (NK) cell activity and receptor expression. **(A)** NK cells were cultured with IL-2 (circle) in the absence or presence of 100 ng/ml (triangle) or 500 ng/ml (cross) PGE2 for 24 h and cytotoxicity was against K562 cells was examined. Bars represent mean ± SD of three independent experiments. **(B)** Expression of NK receptors, including NKp44, NKp30, NKG2D, and TRAIL (filled profiles) and isotype controls (white profiles), as detected by flow cytometry. Numbers indicate mean fluorescence intensity. *Y*-axis shows cell counts. **(C)** Expression of CD107a on the surface of NK cells detected by flow cytometry. Numbers indicate percentage of CD107a^+^ expression. **(D)** IFN-γ level in NK cells analyzed by intracellular staining and flow cytometry. Numbers indicate percentage of positive expression. **(E)** IFN-γ concentration in culture supernatants of NK cells with or without PGE2 treatment, as determined by enzyme-linked immunosorbent assay. Bars represent mean ± SD of three independent experiments. *P < 0.05 and ***P < 0.001. **(F)** Apoptosis of NK cells analyzed by annexin V/propidium iodide staining and flow cytometry.

**Figure 5:**
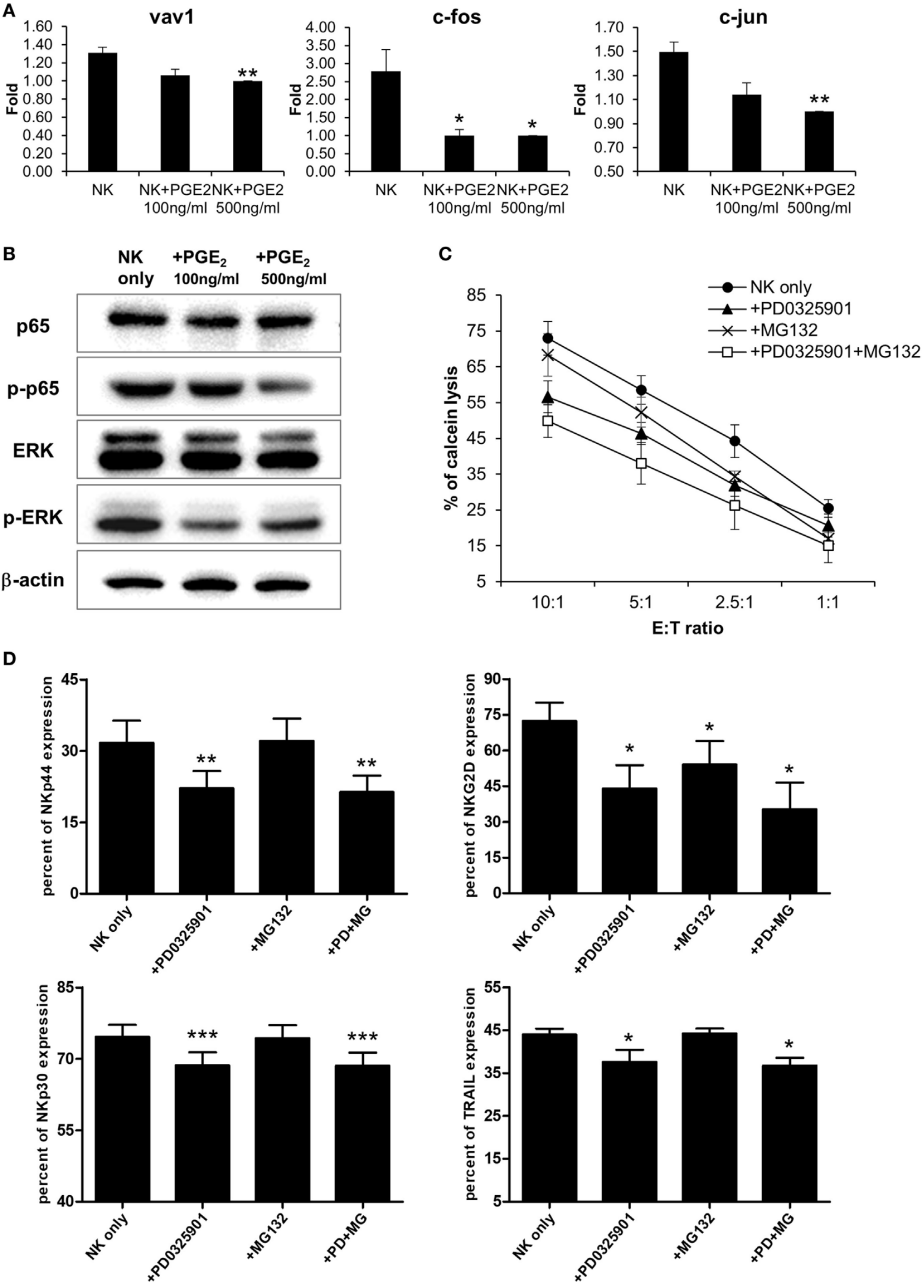
PGE2 inhibits natural killer (NK) cell through extracellular signal-regulated kinase (ERK) and p65 signaling pathway **(A)** mRNA levels of transcription factors related to NK cell functions, as determined by real-time PCR; glyceraldehyde 3-phosphate dehydrogenase served as an internal control. Bars represent mean ± SD of three independent experiments. Statistical analysis was performed using the paired two-tailed Student’s *t*-test. **(B)** Detection of components of signaling pathways regulated by PGE2 in NK cells (including ERK and nuclear factor-κB) by Western blotting; β-actin served as a loading control. **(C)** NK cells were cultured with IL-2 either in control medium (circle) or in medium containing PD0325901 10 µM (triangle), MG132 1 µM (cross), and both PD0325901 and MG132 (white square) for 48 h. The cytotoxicity of NK cells was assessed against K562 cells at different E:T ratio. Bars represent mean ± SD (*n* = 4). **(D)** Percent of receptor expression was analyzed after gating CD56^+^ NK cells by flow cytometry. Bars represent mean ± SD (*n* = 4). Statistical analysis was performed using the paired one-tailed Student’s *t*-test. *P < 0.05, **P < 0.01, and ***P < 0.001.

Natural killer cells are activated by various signals, including mitogen-activated protein kinase (MAPK)/ERK, NF-κB, and signal transducer and activator of transcription (STAT) signals *via* NK activating receptors, cytokines, or external stimuli (25). To identify the pathway through which PGE2 inhibits NK cell activity, NK cells were treated with PGE2. NK cell cytotoxicity was decreased upon treatment with PGE2 relative to untreated control cells (Figure 4A). PGE2 also suppressed the expression of the same NK receptors (NKp44, NKp30, TRAIL, and NKG2D) that were downregulated in NK cells cultured with culture supernatants of thyroid cancer cells (Figures 2F and 4B; Figure S4 in Supplementary Material), and reduced the levels of CD107a, reflecting the degranulation of cytolytic NK cells (Figure 4C). We then examined whether PGE2 affects IFN-γ production, another indicator of NK cell activity. PGE2 treatment suppressed IFN-γ levels in NK cells (Figure 4D). We analyzed IFN-γ levels in culture supernatants of NK cells with or without PGE2 treatment by ELISA and found that IFN-γ secretion was decreased by PGE2, but was restored with co-administration of EP2 receptor antagonist (Figure 4E). We next investigated the effect of PGE2 on NK cell survival by evaluating cell apoptosis. PGE2 did not significantly induce apoptosis in NK cells at concentrations that decreased their activity (Figure 4F). This suggests that decreased NK cytotoxicity by PGE2 is not due to the cell death of NK cells. Therefore, we examined expression of transcription factors involved in other signaling pathways. The mRNA levels of vav-1, c-Fos, and c-Jun were decreased by PGE2 treatment (Figure 5A). These transcription factors are involved in MAPK/ERK signaling and regulate NK cell cytolytic activity and IFN-γ production. We found here that PGE2 reduced the phosphorylation level of not only ERK but also p65 indicating inactivation of NF-κB pathway, which altered vav-1 signaling (Figure 5B). To confirm that reduced ERK and p65 signaling by PGE2 are associated with NK cytolytic activity and NK receptor expressions, NK cells were treated with inhibitors of ERK (PD0325910 10 µM) and p65 (MG132 1 µM). Cytotoxicity of NK cells was decreased by the treatment of each inhibitor compared to that of untreated control, and the NK cytotoxicity was more decreased in NK cells treated with both inhibitors than that of single treatment (Figure 5C). In addition, we have found that expressions of NK receptors related to each signal were different. ERK inhibitor decreased expressions of NKp44, NKp30, NKG2D, and TRAIL, whereas p65 inhibitor decreased expression of NKG2D only (Figure 5D). These findings suggest that PGE2 produced by thyroid cancer cells inhibit NK cell activity by negatively regulating signaling pathways such as ERK and NF-κB.

### PGE2 Inhibits Functional Maturation of NK Cells

**Figure 6:**
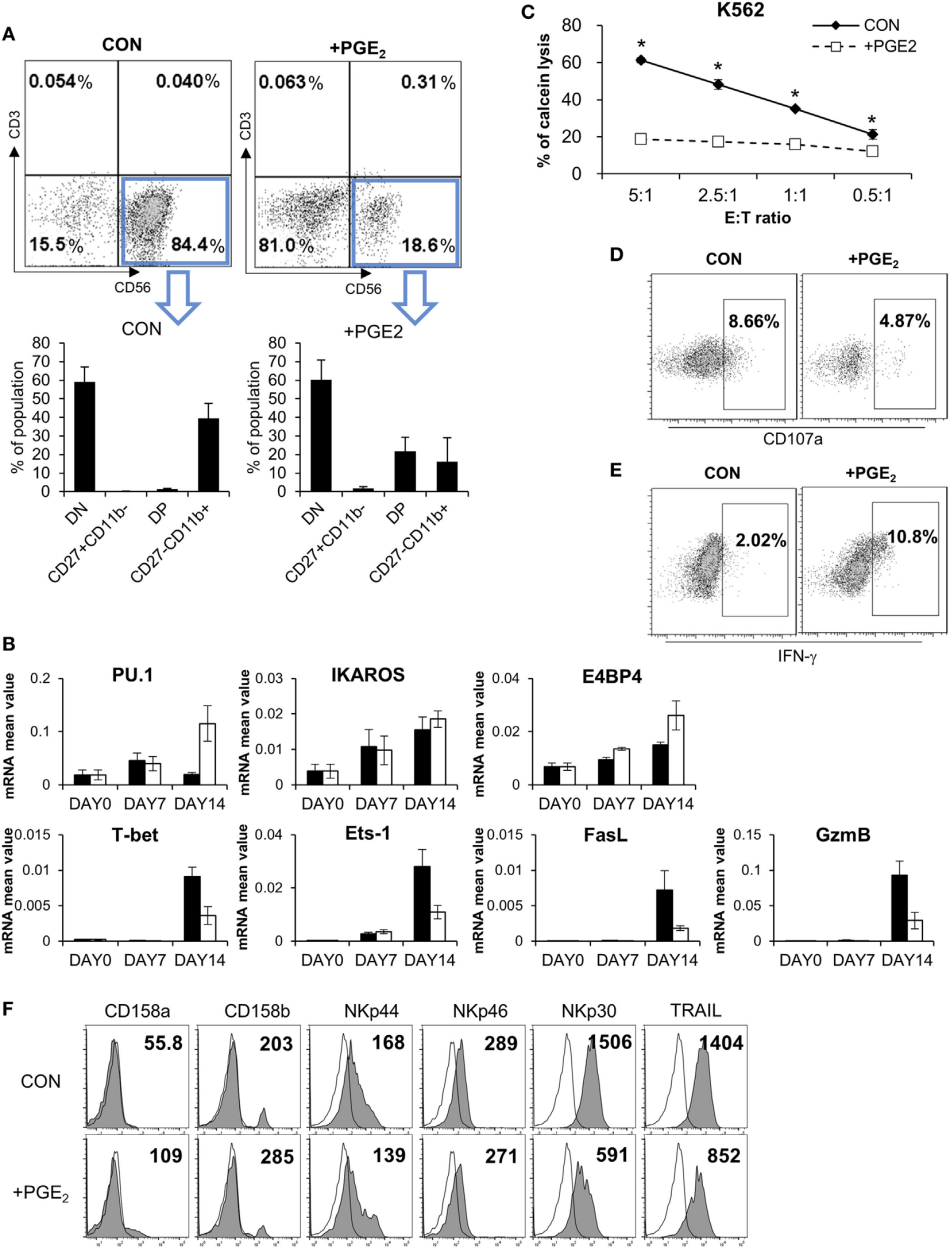
PGE2 inhibits functional maturation of natural killer (NK) cells. **(A)** Flow cytometry analysis of CD27 and CD11b expression in gated CD56^+^ NK cells differentiated from CD34^+^ Hematopoietic Stem Cells. CD56^+^ NK cells were divided into four subsets based on the differential expression of CD27 and CD11b; double negative, CD27^+^CD11b^−^, double positive, and CD27^−^CD11b^+^. **(B)** mRNA levels detected by real-time PCR. Glyceraldehyde 3-phosphate dehydrogenase primer was used as an internal control. **(C)** pNK cells were cultured in complete mNK differentiation medium in the absence (CON: black diamond) or presence of 100 ng/ml of PGE (PGE2: white square) for 14 days; CD56^+^ NK cells were sorted by flow cytometry. Cytotoxicity of NK cells was assessed against K562 cells using calcein-AM. Bars represent mean ± SD of three independent experiments. Statistical analysis was performed using the paired two-tailed Student’s *t*-test (*P < 0.05). **(D)** Expression of CD107a (degranulation marker of NK cells indicating cytolytic ability) was evaluated by flow cytometry. Numbers in the box represent percentage of CD107a^+^ expression. **(E)** IFN-γ expression in NK cells detected by flow cytometry. Numbers indicate percentage of positive expression. **(F)** Analysis of CD158a, CD158b, NKp44, NKp46, NKp30, and TRAIL (filled profiles) expression by flow cytometry. White profiles represent isotype controls. Numbers indicate mean fluorescence intensity. *Y*-axis shows cell counts.

Although PGE2 is known to suppress the differentiation of cytotoxic T cells, dendritic cells (DCs), and type 1 T helper cells (Th1) (26–28), there is little known regarding the effect of PGE2 on NK cell differentiation. We established a system for inducing the differentiation of NK cells from CD34^+^ HSCs derived from CB (29, 30), which included pNK and mNK cells. To confirm the effect of PGE2 on NK cell differentiation, pNKs were treated with PGE2. This decreased the fraction of CD56^+^ cells (18.6%) relative to untreated control cells (84.4%), indicating that PGE2 prevented the transition of pNK to mNK cells (Figure 6A, top). CD56^+^ NK cells were divided into four subsets based on the differential expression of CD27 and CD11b, such as CD27CD11b^−^, CD27^+^CD11b^−^, CD27^+^CD11b^+^, and CD27^−^CD11b^+^ (31). In particular, it has been reported that the expression of CD27 in human NK cells is related to the functional maturation of NK cells; the absence of CD27 expression marks the maturation of NK cells to effector cell phenotype (32, 33). When CD56^+^ NK cells were gated, cells treated with PGE2 during differentiation had a lower percentage of functionally mature (CD27^−^CD11b^+^) cells than untreated control NK cells (Figure 6A, bottom). Since NK cells express specific genes at each stage of differentiation, we evaluated the mRNA expression of genes related to NK cell development (Figure 6B). Cells treated with PGE2 expressed PU.1 and E4 promoter-binding protein, which are transcription factors that are expressed in immature NK cells. On the other hand, genes expressed in mature NK cells, such as T-bet, Ets-1 (34, 35), and those related to cytolytic function including Fas ligand (FasL) and granzyme B were expressed at a higher level in untreated as compared to PGE2-treated NK cells.

Since control and PGE2-treated NK cells had different rates of NK cell differentiation, we sorted CD56^+^ NK cells and performed a cytotoxicity assay to evaluate NK cell activity under different conditions. PGE2 treatment significantly reduced NK activity as compared the control group (Figure 6C). We measured CD107a and IFN-γ levels after gating CD56^+^ NK cells by flow cytometry and found that CD107a expression—which reflects NK cell activity—was decreased by PGE2 treatment (Figure 6D), whereas IFN-γ expression showed the opposite trend (Figure 6E). These results suggest that treatment of PGE2 impaired functional maturation of NK cells, releasing more IFN-γ and showing less cytolytic activity than mature NK cells. Expression of NK activating receptors—especially NKp30 and TRAIL—in CD56-gated NK cells was decreased upon PGE2 treatment during NK cell differentiation. In addition, PGE2 increased the expression of inhibitory receptors such as CD158a (KIR2DL1) and CD158b (KIR2DL3) (Figure 6F). These results indicate that PGE2 suppresses NK cell activity through downregulation of NK activating receptors and upregulation of NK inhibitory receptors. Thus, PGE2 affects not only the activity of mature NK cells but also functional maturation of immature NK cells.

## Discussion

In this study, we demonstrated that thyroid cancer cells inhibit the function of NK cells by secreting PGE2, which inhibits the expression of NK activating receptors such as NKp44 and NKp30 and TRAIL, a death receptor ligand. We also found that PGE2 inhibits the differentiation and maturation of NK cells.

Natural killer cells have several activating receptors, including NKG2D, NKp46, NKp44, and NKp30 that recognize their cognate ligands on target cells. Cytotoxicity is induced by binding of activating receptors on the NK cell surface to ligands on target cells. Therefore, high levels of NK receptor ligands on target cells could affect NK cell-mediated killing of thyroid cancer cells. However, NK cells showed poor cytotoxicity toward ATC cells despite the high expression of NKp44, NKp30, and NKG2D ligands on the latter (Figure S5A in Supplementary Material). This suggests that expression of NK receptor ligands on target cells is not the sole determinant of cytolytic activity and that other factors protect ATC cells from NK cells.

Although NK cells can kill thyroid cancer cells, thyroid cancer cells also affect NK cells through factors secreted into the tumor microenvironment (1, 2, 36–38), including PGE2, IDO, and TGF-β, among others. Since thyroid cancer cells show variable resistance to NK cell cytotoxicity depending on tumor stage (Figure S5B in Supplementary Material), we searched for a factor that enhanced resistance to NK cells. COX-2 is upregulated in various cancers including thyroid cancer (39–42). Our results showed that ATC cells released larger amounts of PGE2 and suppressed NK cell activity to a greater extent than PTC cells (Figures 2–4). Unlike our results, Wennerberg et al. reported that the susceptibility of ATC to NK cells was higher than PTC because of the differences in the expression level of NKG2D ligands and chemokine levels (43). They have shown the differences in sensitivity to NK cells against thyroid cancer cell lines derived from ATC and PTC patients but we used PTC (TPC-1 and BCPAP) and ATC cell lines (FRO and 850-5c) that have been generally used and experimentally verified. It is possible that the differences in susceptibility to NK cells may have been attributed by the different cell lines that we used. However, the expression level of PGE2 is higher in ATC patients than PTC patients resulting in poor function of NK cells, which is consistent with our results that NK activity was suppressed by PGE2 in thyroid cancer cells. In addition, our results showed that the expression levels of COX-2 and PGE2 are significantly increased in ATC cell lines compared to PTC cell lines (Figure 2; Figure S3 in Supplementary Material). This is supported by Ji et al. who observed that COX-2 was more expressed in ATC patients than in PTC patients by immunochemistry suggesting that high expression of COX-2 is correlated with thyroid cancer with poor prognosis (44). PGE2 is a metabolite of COX-2 that regulates cancer cell proliferation, survival, migration, and invasion. PGE2 stimulates cancer cell proliferation *via* multiple cascades and a glycogen synthase kinase-3β–β-catenin signaling or through upregulation of aromatase (45). It also promotes cancer cell survival by activating the phosphoinositide 3-kinase (PI3K)–Akt–peroxisome proliferator-activated receptor-δ cascade (46–48). In addition, PGE2 induces colorectal cancer cell migration and invasion through β-arrestin-1–SRC–epidermal growth factor receptor–PI3K–Akt signaling (49, 50). Thus, cancer cells protect themselves *via* PGE2-dependent mechanisms. On the other hand, PGE2 can undermine immune responses by suppressing the cytotoxic function of NK cells and cytotoxic T lymphocytes, suppressing the production of IFN-γ by immune cells, and disrupting the differentiation of DCs, Th1, and cytotoxic T lymphocytes (26). Our data also showed that PGE2 produced by thyroid cancer cells blocks NK cell-mediated cytotoxicity (Figure 2), suggesting that targeting the COX-2/PGE2 pathway in thyroid cancer may be an effective therapeutic strategy.

PGE2 enters cells through the G-protein coupled receptors EP1–4, which are structurally similar but are associated with different intracellular signaling pathways (51). NK cells differentiated from peripheral blood expressed EP2 and EP4 receptors and the expressions of EP receptors in NK cells cultured in thyroid cancer cell supernatant were increased. This indicates that PGE2 in thyroid cancer cell supernatant upregulated the expressions of EP2 and 4 receptors in NK cells. We used specific antagonists to determine whether PGE2 released from thyroid cancer cells affects NK cells through EP receptors and found that this treatment restored the inhibition of NK cytotoxicity induced by TPC-1 and 850-5C thyroid cancer cell supernatant to varying degrees (Figure 3). These results demonstrate that PGE2 decreases NK cell activity through EP2 and EP4 receptors.

Activation of NK activating receptors through MAPK kinase 1/2 and ERK1/2 pathways increase cytotoxicity and IFN-γ secretion (52, 53). In our study, treatment of NK cells with PGE2 reduced NK cytotoxicity and the expression of NK receptors and transcription factors related to NK activation (Figures 4 and 5). The amount of PGE2 in 850-5C thyroid cancer cells was over 1,000 pg/ml (Figure 2). We determined that NK cytotoxicity was decreased in a PGE2 concentration-dependent manner (Figure S6 in Supplementary Material). Vav-1, a downstream effector of NK activating receptors, mediates NK cell cytotoxicity through MAPK/ERK and NF-κB pathways (54). C-Fos and c-Jun are transcription factors stimulated by activated NK cells under NK activating receptor or cytokine receptor signaling, which occurs through the MAPK/ERK or STAT3/STAT4 pathway or NF-κB signaling, which increases IFN-γ production (52). PGE2 suppressed the phosphorylation of NF-κB and MAPK/ERK, resulting in the downregulation of the transcription factors vav-1, c-Fos, and c-Jun. In addition, we confirmed that suppression of ERK and p65 signaling induced by PGE2 is involved in NK cytolytic activity and NK receptor expressions by the treatments of inhibitor of ERK and p65. We found that both inhibitors reduced NK cytolytic activity and NK receptor expression although ERK inhibitor reduced expression of NK activating receptors, including NKG2D, NKp44, and NKp30, but p65 inhibitor reduced that of NKG2D receptor only (Figure 5). Taken together, these suggest that PGE2 suppresses NK cells activation by inhibiting NF-κB and MAPK/ERK signaling.

To investigate the effect of PGE2 on NK cell differentiation, we sorted HSCs from mononuclear cells in CB and induced their differentiation into NK cells with appropriate media. PGE2 inhibited NK cell functional maturation (Figure 6). NK cells in control media without PGE2 showed higher numbers of CD11b^+^CD27^−^ cells with relatively high cytolytic activity; however, in the presence of PGE2, there was an increase in the CD11b^+^ CD27^+^ fraction, which have lower cytolytic activity and a higher capacity for cytokine secretion than the CD11b^+^CD27^−^ subset. This may be related to the low expression of activating receptors and high expression of inhibitory receptors in NK cells treated with PGE2 (Figure 6F). Interestingly, NK cells differentiated in medium containing PGE2 produced more IFN-γ than control cells. We speculate that PGE2 maintains NK cells in a functionally immature state that is less cytotoxic. This was substantiated by our observation that NK cells differentiated in medium containing PGE2 expressed lower levels of granzyme B and FasL that those induced to differentiate in the absence of PGE2 (Figure 6).

In conclusion, PGE2 produced by thyroid cancer cells suppressed the cytolytic activity of NK cells by inhibiting the expression of NK activating receptors through EP2 and EP4 receptors on NK cells. In addition, PGE2 inhibited functional maturation and cytolytic activity of NK cells. Moreover, a more advanced type of thyroid cancer (850-5C) released higher amounts of PGE2, suggesting a correlation between PGE2 production and thyroid cancer progression. Thus, PGE2 secreted by cancer cells may have a protective function, allowing cancer cells to evade immune surveillance and the cytolytic activities of NK cells in the tumor microenvironment.

## Ethics Statement

This study was approved by the Institutional Review Board of the Asan Medical Center according to the Bioethics and Safety Act and the Declaration of Helsinki. Each participant provided written, informed consent.

## Author Contributions

AP performed experiments, analyzed data, and drafted the manuscript. MK, YL, and YK assisted with some experiments. YP, HJ, TK, and HL provided useful suggestions. IC and SY supervised the study, helped to design the experiments, and wrote the manuscript.

## Conflict of Interest Statement

The authors declare that the research was conducted in the absence of any commercial or financial relationships that could be construed as a potential conflict of interest.
