# Supplementary material for: Prostaglandin E2 Secreted by Thyroid Cancer Cells Contributes to Immune Escape Through the Suppression of Natural Killer (NK) Cell Cytotoxicity and NK Cell Differentiation
Source: Front Immunol. 2018 Aug 9;9:1859. doi: 10.3389/fimmu.2018.01859 (PMC6094168; doi:10.3389/fimmu.2018.01859)
Supplement: Supplementary file 1 [file data_sheet_1.docx]

Supplementary Material

Prostaglandin E2 secreted by thyroid cancer cells contributes to immune escape through the suppression of NK cell cytotoxicity and NK cell differentiation

Arum Park1,2, Mi Sun Kim1, Yunhee Lee3, Young Ju Kang 4, Young-Jun Park1,2, Haiyoung Jung1,2, Tae-Don Kim1,2, Hee Gu Lee1,2, Inpyo Choi1,2* and Suk Ran Yoon1,2*

*** Correspondence:** Suk Ran Yoon, Ph. D or Inpyo Choi, Ph. D

Immunotherapy Convergence Research Center, Korea Research Institute of Bioscience and Biotechnology, 125 Kwahak-ro Yuseong-gu, Daejeon 34141, Republic of Korea

Tel: +82-42-860-4239, Fax: +82-42-860-4593

e-mail: [sryoon@kribb.re.kr](mailto:sryoon@kribb.re.kr) or [ipchoi@kribb.re.kr](mailto:ipchoi@kribb.re.kr)

# Supplementary Figures

**Supplementary Figure 1.** Changes in expression of NK cell receptors by thyroid cancer supernatant and effects of PGE2 inhibitor. Expressions of NK activating receptors and death receptors were analyzed by flow cytometry. (A) N=7. (B) N=4. Statistical analyses were performed using the paired one-tailed Student’s t test. (*, P < 0.05; **, P < 0.01; ***, P < 0.001) Horizontal lines within graph represent median value


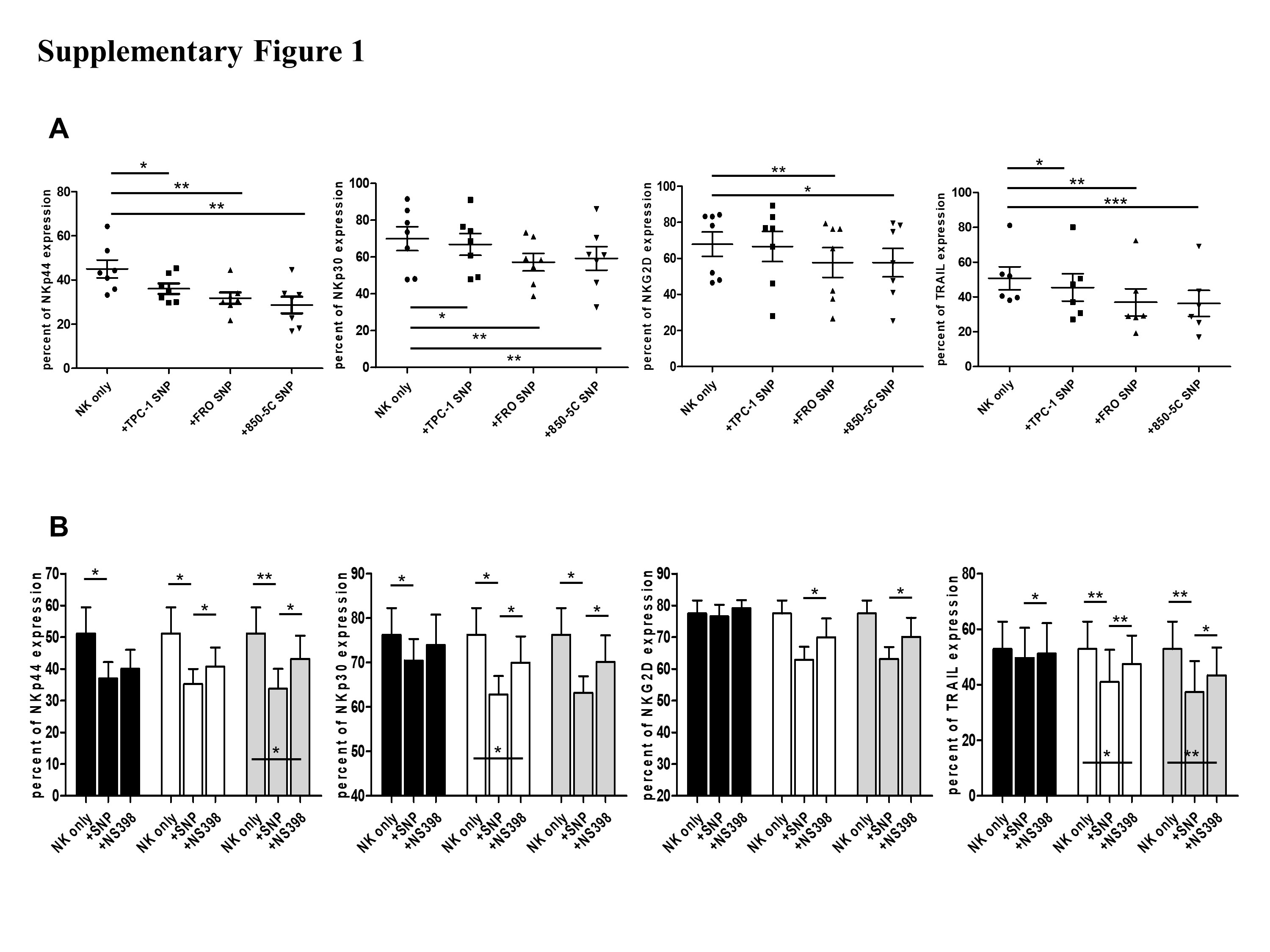

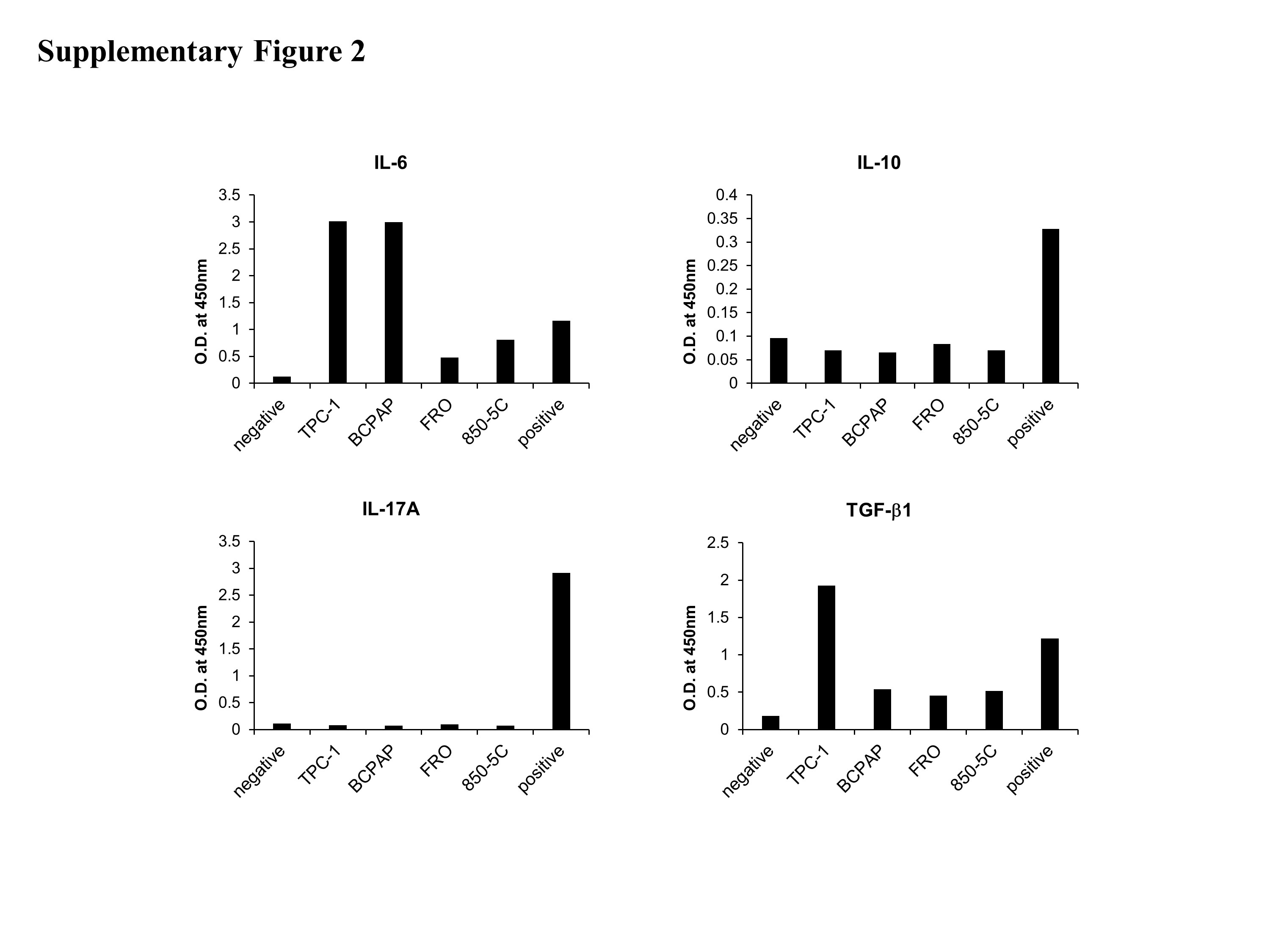


**Supplementary Figure 2.** Cytokines in culture **supernatants** from thyroid cancer cells. Production of IL-6, IL-10, IL-17A and TGF-β1 from four types of thyroid cancer cells supernatants cultured for 72hours was analyzed by ELISA array (QIAGEN). Absorbance was read at 450 nm.


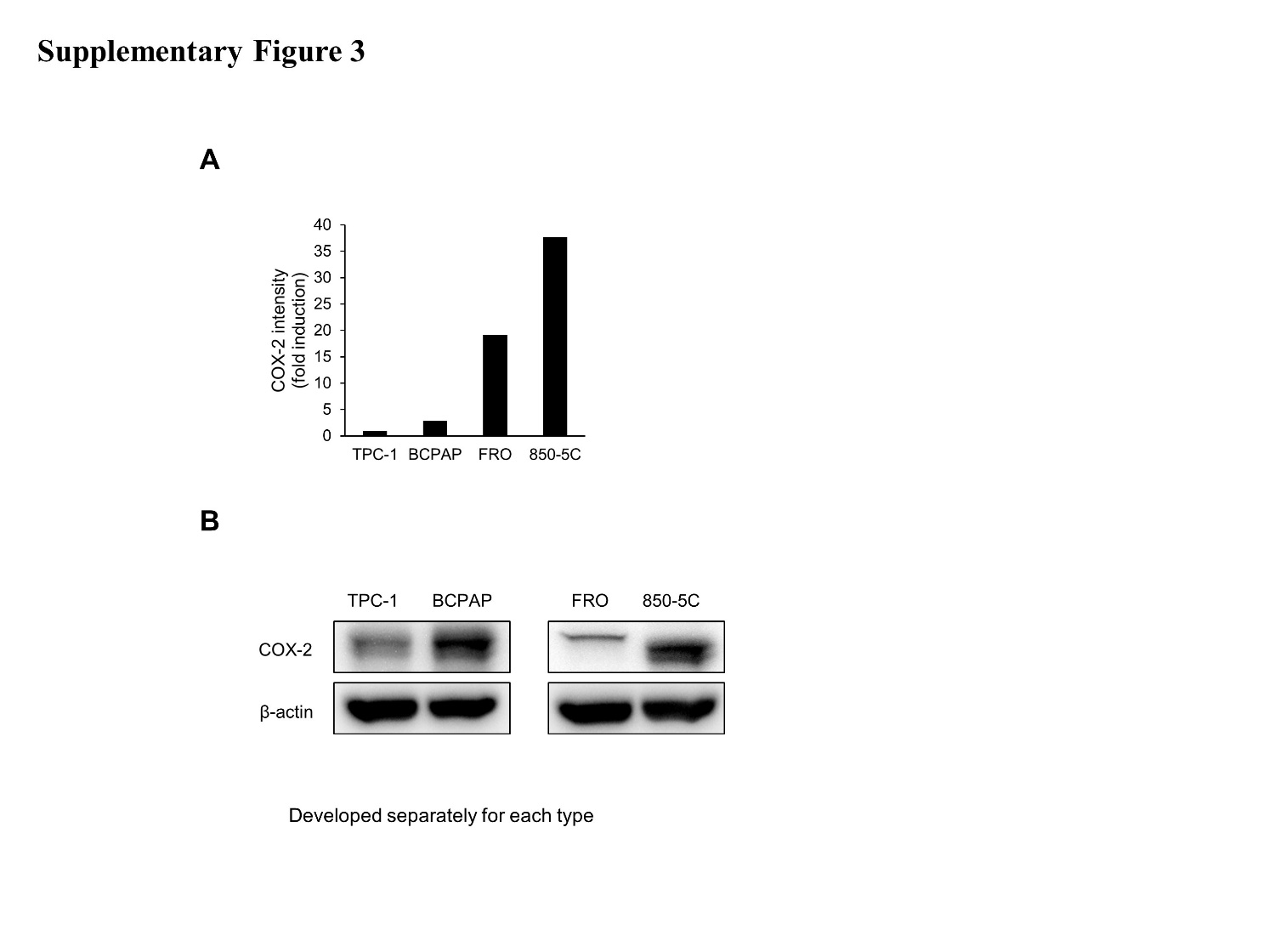
**Supplementary Figure 3.** COX-2 is expressed in both papillary (TPC-1, BCPAP) and anaplastic (FRO, 850-5C) thyroid cancer cells. (A) Comparison of intensity differences in COX-2 expression in four cell lines. (B) When papillary and anaplastic thyroid cancer samples were separately developed with different exposure time (3 mins for papillary thyroid cancer cells and 10 sec for anaplastic thyroid cancer cells). COX-2 band was clearly seen in the papillary cancers as well as in anaplastic cancer . β-actin was used as a loading control.


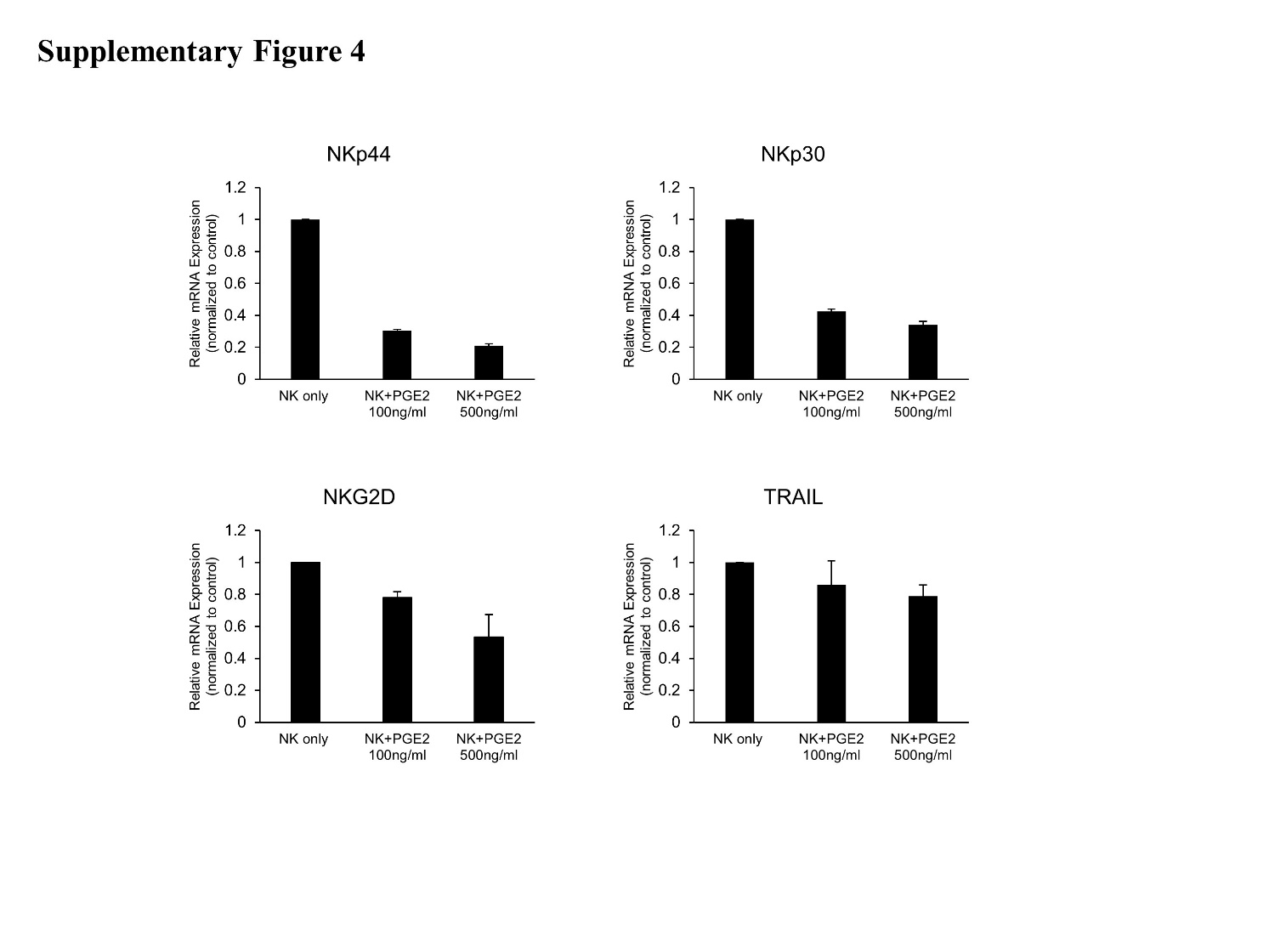
**Supplementary Figure 4.** PGE2 also down regulated NK receptor expression at transcription level. mRNA levels were detected by real-time PCR. GAPDH primer was used as an internal control.


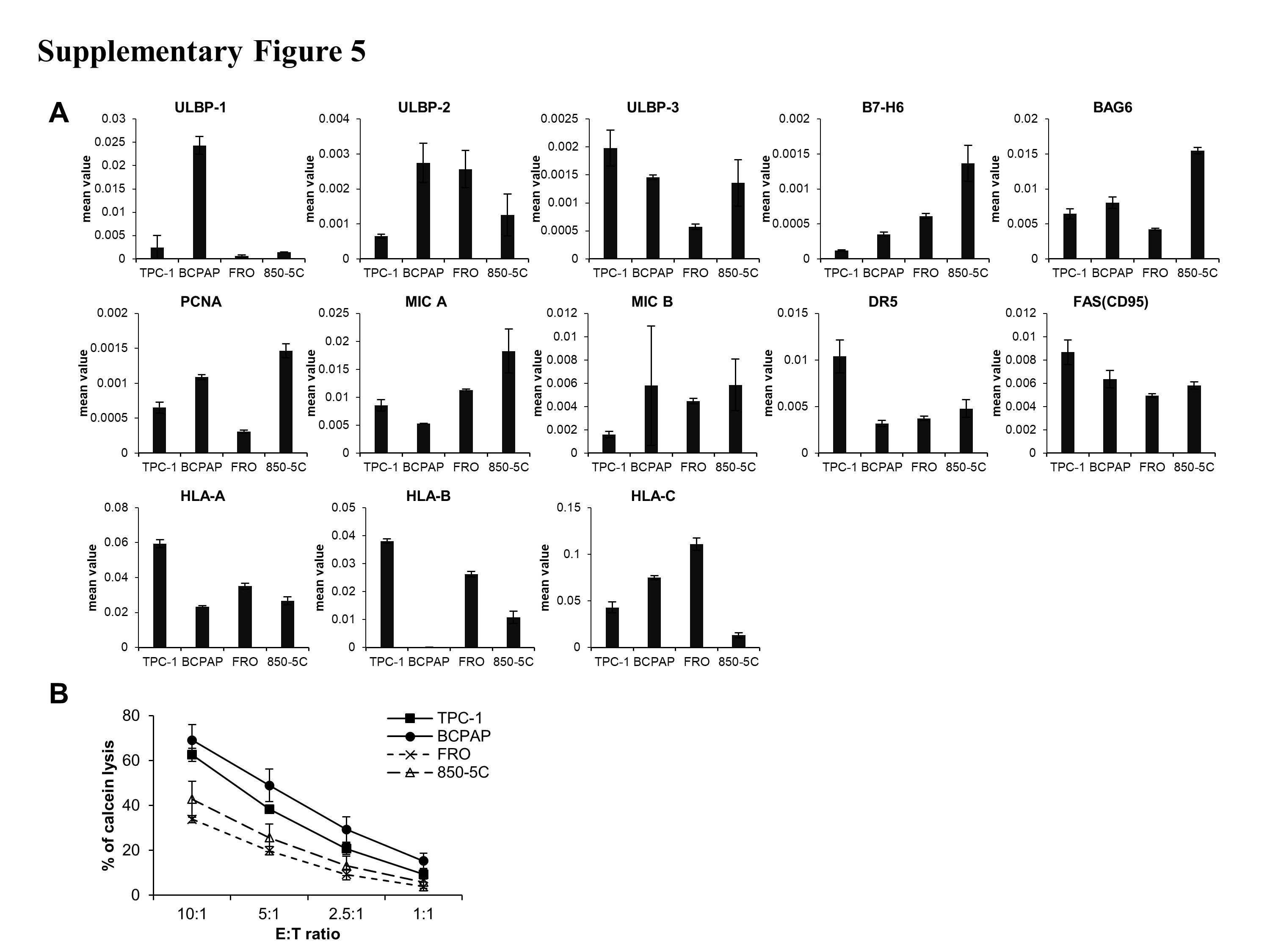


**Supplementary Figure 5.** Thyroid cancer cell lines showed different susceptibility to NK cell cytotoxicity. (A) The mRNA expression of ligands that bind to NK activating receptors on four type of thyroid cancer cells were analyzed **by real-time** PCR. GAPDH primer was used as an internal control. Bars represent means ± SD obtained from three independent experiments. (B) Cytotoxicity of NK cells was assessed against the indicated thyroid cancer cells by calcein-AM assay. Bars represent means ± SD obtained from three independent experiments.


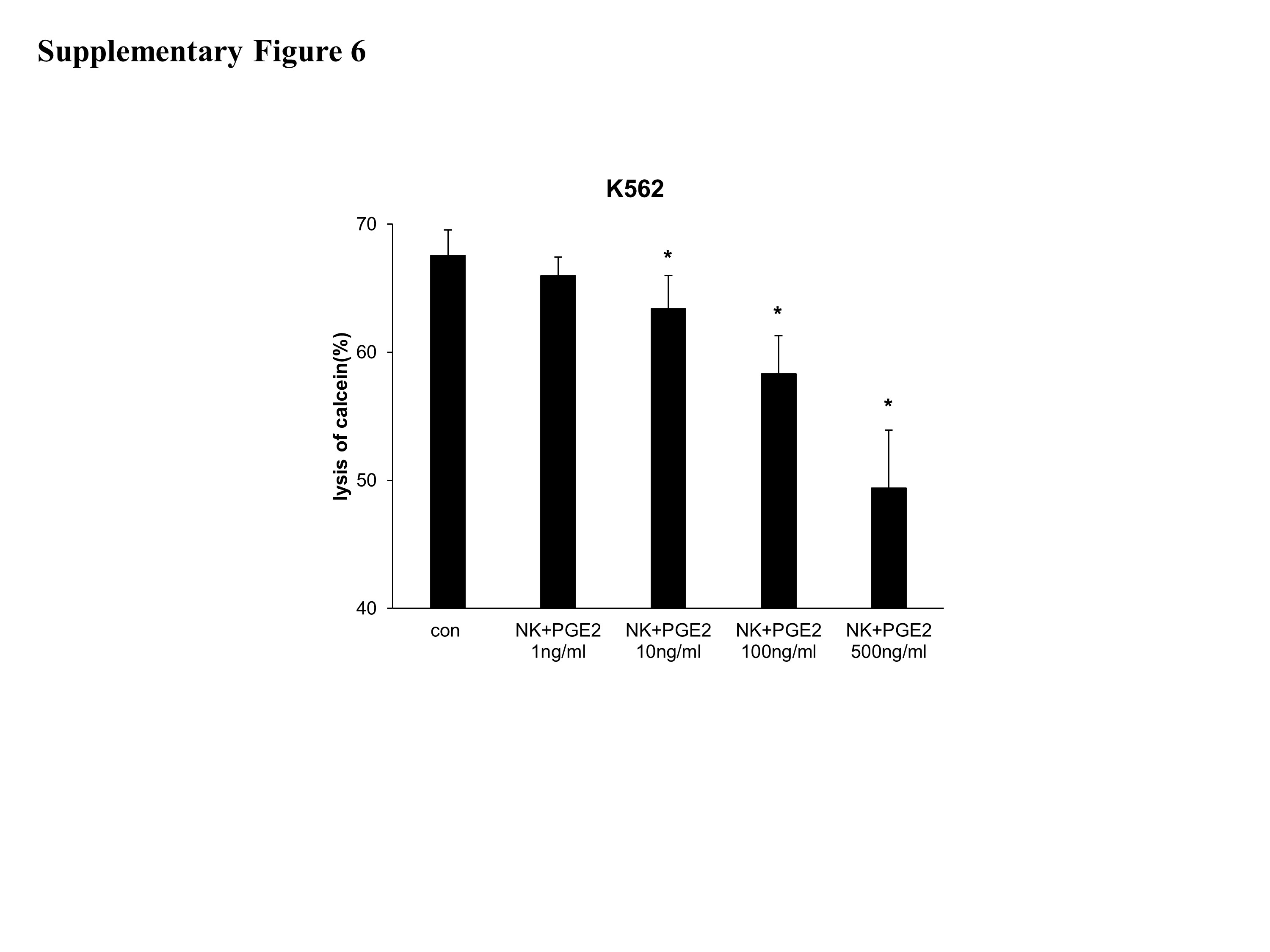


**Supplementary Figure 6.** PGE2 down regulates NK activity in a dose dependent manner. NK cells were cultured with various concentration of PGE2 for 24hours and cytotoxicity of the NK cells was assessed by calcein-AM assay using K562 as target cells at 5:1 of E: T ratio. Bars represent means ± SD obtained from three independent experiments. ***P < 0.05**
